# Effectiveness of antenatal yoga in reducing intensity of labour pain: A systematic review and meta-analysis

**DOI:** 10.1016/j.eurox.2023.100214

**Published:** 2023-07-05

**Authors:** Deenadayalan Boopalan, Venugopal Vijayakumar, Poornima Ravi, Poonguzhali shanmugam, Bincy Kunjumon, Maheshkumar Kuppusamy

**Affiliations:** a Senior Research Fellow, National Institute of Mental Health and Neurosciences, Bengaluru, Karnataka, India; b Department of Yoga, Govt. Yoga & Naturopathy Medical College & Hospital, The Tamilnadu Dr. MGR Medical University, Chennai, India; c Senior Research Fellow, Sri Ramachandra Institute of Higher Education and Research. Chennai, India; d Department of Community medicine, Govt. Yoga & Naturopathy Medical College & Hospital, The Tamilnadu Dr. MGR Medical University, Chennai, India; e Department of Community Medicine, SRM Medical College Hospital and Research Centre, SRMIST, Chennai, India; f Department of Physiology, Govt. Yoga & Naturopathy Medical College & Hospital, The Tamilnadu Dr. MGR Medical University, Chennai, India

**Keywords:** Yoga, Antenatal yoga, Delivery outcome, Pain, Meta-analysis

## Abstract

**Background:**

Yoga during pregnancy was found to be beneficial in various aspects of pregnancy including pain management during the time of labour. The current systematic review and meta-analysis aims to assess the effectiveness of antenatal yoga practices in reducing pain during the time of labour.

**Methods:**

We searched electronic databases such as PubMed, Embase, Cochrane Library, and Web of Science, till January 2023. Randomized controlled trials (RCTs) which measured the effects of antenatal yoga practices on pain management during labour were included. The main outcome was the pain intensity measured with any validated questionnaire. The methodological quality of included studies was evaluated by using a risk-of-bias assessment tool developed by the Cochrane Collaboration. For the effect size, standardized mean differences (SMDs) with a 95% confidence interval (CI) were generated with a random effect model using R software (version 4.2.2).

**Results:**

Eight studies including 576 antenatal women between the age of 14 and 40 years were included. Results of this meta-analysis showed that yoga is effective in reducing labour pain (SMD: −1.34 95% of CI: −1.86, −0.81) with significant heterogeneity among the studies (I^2^ 73%, p < 0.0008).

**Conclusion:**

Antenatal yoga can be a promising intervention in the field of obstetrics to reduce the intensity of labour pain. However, we are still in need of RCT with a large sample size to confirm the reliability of the present meta-analysis.

## Background

Labour is a normal physiological process of delivering a baby to the external world and the pain experienced by the women during labour and childbirth is a complex phenomenon [1]. Pain is a unique individual experience, and the pain experienced by women varies widely depending upon the sensory and affective perception [2]. Labour pain is a combination of unpleasant sensory, perceptual, and emotional experiences which is associated with autonomic, physiological, emotional, and behavioural responses [3]. Labour usually involves three stages, namely, effacement and dilatation of the cervix, delivery of baby, and delivery of placenta and recovery (1–4 h postpartum). Pain originates from different sites during labour which includes uterine contraction, dilatation of the cervix and stretching of pelvic floor muscles. Pain experienced by women during labour is influenced by maternal position, anxiety, fear, stress, pain tolerance, and social support [4]. Several pharmacological and non-pharmacological interventions were used effectively to manage pain in labour. Pharmacological management for labour pain involves the usage of opioid drug, epidural, inhaling analgesic and combined spinal epidurals. However, pharmacological interventions are associated with adverse effects such as nausea, vomiting and dizziness. At times, it may also lead to incidences of instrumental delivery, fetal distress, caesarean section, urinary incontinence, motor nerve blockade, hypotension and fever [3]. Available evidences have documented that the non-pharmacological interventions such as, breathing exercises, relaxation techniques and massage reduce labour pain intensity during the active phase of labour [5]. Similarly, other non-pharmacological interventions such as essential oils, acupressure, music therapy, aromatherapy, perineal massage [6] and yoga are commonly used to manage labour pain as well [7], [8], [9]. Stress and anxiety are common problems faced during pregnancy, which could directly impact the course of pregnancy and even labour. Mind-body interventions such as hypnotherapy, tai-chi, auto-suggestion, meditation, reflexology [10] and yoga are proven to be beneficial in the management of anxiety during pregnancy [7]. Yoga is a widely used mind body intervention technique, gaining attention in recent times, concerned to its beneficial effect during pregnancy and labour. Yoga not only comprises of asanas, but also pranayama, mudra, meditation and also relaxation practices that are documented to be useful during pregnancy [11]. Child birth is associated with stress response and production of catecholamine affects labour process [12]. Yoga is documented to be beneficial in reducing pain and stress while also preparing the mind of pregnant women for labour. Although the recommendation of yoga for antenatal care and labour ease has been reported previously, there is a need for a comprehensive assessment. Hence, the objective of the present systematic review and meta-analysis is to evaluate the impact of antenatal yoga in reducing labour pain.

## Methodology

### Data sources and searches

For the current systematic review and meta-analysis, PubMed, Embase, Cochrane Library and Web of Science, CINAHL and Google Scholar were systematically searched for the article published from inception through January, 2023. The following medical subject heading term (Mesh) and key words are used: “Yoga” [Mesh], “Yoga” OR “Prenatal Yoga” AND “labour pain”[Mesh], “Labour Pain” OR “Delivery Pain”. Only articles published in English language is included for the meta-analysis. In addition, references from included studies and pertinent review articles were searched to identify other studies meeting the selection criteria. The present systematic review and meta-analysis was conducted and reported as per the Preferred Reporting Items for Systematic Reviews and Meta-Analyses (PRISMA) guidelines [13].

### Study selection

Two authors (DB, PR) independently identified articles eligible for review and meta-analysis. Randomized controlled trials (RCTs) examining the effects of antenatal yoga practices on pain management during labour were included. Articles that met the following (PICO) criteria were screened.

(1) Participants: Pregnant women with age above (≥14 years).
(2) Intervention: Yoga is the primary intervention, which includes components such as asanas, pranayama, relaxation techniques, Om chanting, and meditation.
(3) Comparison: Pregnant women receiving standard care or any other interventions apart from yoga.
(4) Outcome: The studies that assessed labour pain as an outcome measure were included.

### Exclusion criteria

Participants with twin pregnancy, gestational hypertension, gestational diabetes mellitus and high-risk pregnancy were excluded. Articles written in languages other than English were also excluded. Studies using other interventions or therapies in addition to yoga were not included.

### Data extraction and quality assessment

Data was independently extracted by two authors (DB, PR) using a standardized protocol and reporting form. Disagreements were resolved by arbitration, and consensus was reached after discussion. The following information was extracted: study characteristics (study name, authors, publication year, Country of origin, sample size, study design, intervention details, outcome measures, and findings), study sample characteristics (mean age and sex), main exposure (antenatal yoga) and main outcome (Visual analogue scale (VAS) - labour pain). Two investigators assessed (DB and PR) the methodological quality of the included studies as recommended by the Cochrane handbook of assessing risk of bias in a randomized trial. Domains such as random sequence generation, allocation concealment, blinding of participants and personnel reporting, blinding of outcome assessment, other sources of bias, incomplete outcome and data selective outcome is assessed in all the included studies [10].

### Statistical analysis

The mean (i.e.) VAS differences between experimental and control groups were estimated. Standardized mean difference (SMD) and 95% confidence interval (CI) were calculated to determine the effect size using random effect model. The I^2^ statistics and Cochran Q test was used to assess heterogeneity. Analysis was done with metafor package in R statistical software version 4.2.2.

## Results

### Literature selection

**Fig. 1:**
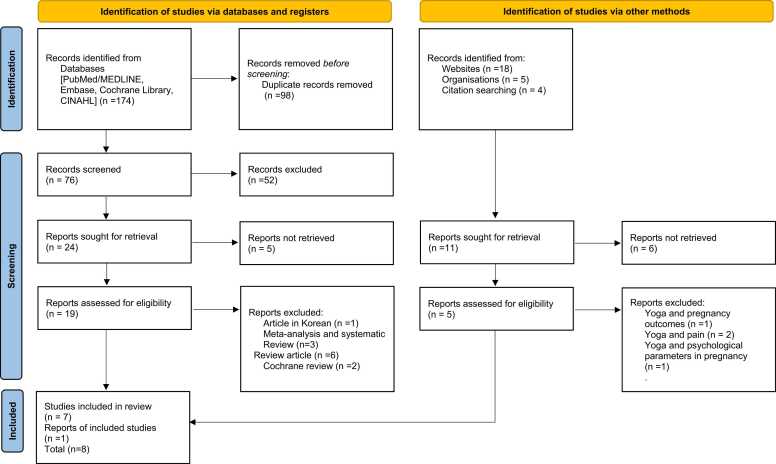
PRISMA flow chart of study selection.

We identified 201 potential articles from the initial literature search. After reading title, abstract and removing duplicates, 178 articles were excluded by three independent authors. Of those, 23 potential articles were retrieved after carefully reading through the full text, out of which 8 articles were included for review and 7 articles were included for the meta-analysis (Fig. 1).

### Literature characteristics

Table 1 shows the literature characteristics of the included studies. Out of 8 studies, two studies [14], [15] was done in Indonesia, two studies [16], [17] in Iran, rest in India [18], [19], Thailand [20] and Brazil [21]. Out of those eight included studies, six studies [14], [16], [17], [18], [19], [20] done on primigravida mothers. The age of the participants ranges from 14 to 40 years. Maximum sample size was 75 in both study and control group, while the minimum sample size was 11 in both groups. Intervention of study group involved practice of asanas, pranayama, dhyana, relaxation, Om chanting, and Yoga nidra. Minimum duration of intervention was 30 min and maximum duration was 1 h. Most commonly used parameters for assessment in all the studies was visual analogue scale.

**Table 1:** Characteristics of included studies.

First Author (Year)	Country	Study Design	Participants with sample size	Intervention details	Outcome measures	Findings
Chuntharapat et al., 2008 [18]	Thailand	Randomized Controlled Trial	18 − 35 years primigravid women 66 (Yoga group-33; control group-33)	Yoga asanas, chanting om, breathing awareness, yoga nidra, and dhyana 60-min practice sessions at the26–28th, 30th, 32nd, 34th, 36th, and 37th week of gestation.	State-Trait Anxiety Inventory; The visual analogue scale total comfort (VASTC); Maternal comfort questionnaire (MCQ); The visual analogue sensation of pain scale(VASPS); The pain behavioural observation scale (PBOS)	Practice of 30 mins of yoga, at least thrice for 10 weeks facilitates in maternal comfort, decreasing pain during labour, and 2 h post-delivery and shortening length of labour
Martins et al., 2014 [19]	Brazil	Randomized Controlled Trial	14–40 years of age, 12–32 weeks of gestation. yoga group −21 and control group −24	asanas, breathing, meditation and relaxation	VAS, lumbar pain provocation test, posterior pelvic pain provocation test	yoga decreased pain intensity in lumbar and posterior pelvic region
Jahdi et al., 2017 [15]	Iran	Randomized Controlled Trial	sixty primiparous women, aged 18–35 years	1-hour supervised yoga class, three times a weekly, starting at 26 weeks gestation.	Labour pain and discomfort level measured using a Visual Analogue Scale	Yoga during pregnancy may reduce pain of labour and improved adequacy of childbirth.
Karnasih 2018 [13]	Indonesia	Randomized Controlled Trial	22 (Yoga group-11; control group-11)	asanas, chanting om, dhyana, yoga nidra and breathing awareness	VAS at 2–4 cm of cervical dilatation	In yoga group-duration of second and third stage of labour was decreased, labour pain score decreased, C- section decreased
Bolanthakodi et al., 2018 [16]	India	Randomized Controlled Trial	150 (Yoga group-75; control group-75) primigravida, 20–35 years of age, gestational age of 30weeks	30-min practice sessions at 30th, 32nd, 34th, 36th, 37th, 38th, and39th weeks	Alleviation of labour pain was assessed by using numerical pain intensity scale(NPIS), pain behaviour observational scale(PBOS),and maternal delivery comfort questionnaire	in yoga group decrease in oxytocin augmentation, intravenous analgesics, in number of vaginal deliveries, length of labour shortened, tolerance of pain is better in study group
Mohyadin et al., 2021 [14]	Iran	Randomized Controlled Trial	84 (Yoga group-42; control group-42) primigravida, above 18 years of age.	six 60-min training sessions for every 2 weeks from week 26 of pregnancy and continued until 37 weeks of gestation.	State-Trait Anxiety Inventory, labour pain - Visual Analogue Scale (VAS)	Practicing yoga during pregnancy mayreduce women’s anxiety during labour
Rahayu et al., 2023 [12]	Indonesia	Non-randomized experimental study	59 primigravida females (Yoga group-30; control group-29) with 20–35 years of age.	modified Iyengar yoga weekly once with 90 min of duration for three months	Degree of anxiety- Hamilton Scale Rating for Anxiety (HSRA) labour pain - Visual Analogue Scale (VAS)	Iyengar yoga in primigravida women was beneficial in reducing labourpain and anxiety.
Esencan and Rathfisch (2023)[19]	Turkey	Randomized Controlled Trial	90 (Yoga group-30; control group-60) primiparous pregnant women	yoga and meditation for 60 min two times a week for 10 weeks.	State Trait Anxiety Inventory (STAI) Wijma Delivery Expectancy/Experience Questionnaire, Childbirth Self-Efficacy Scale (CBSEI) Short Form Wijma Delivery Expectancy/Experience Questionnaire Version B, visual analogue scale (VAS) for pain.	Yoga and meditation are effective methods for reducing pain and fear perception and increasing self-efficacy and vaginal delivery rates during the labour

### Meta- analysis

**Fig. 2:**
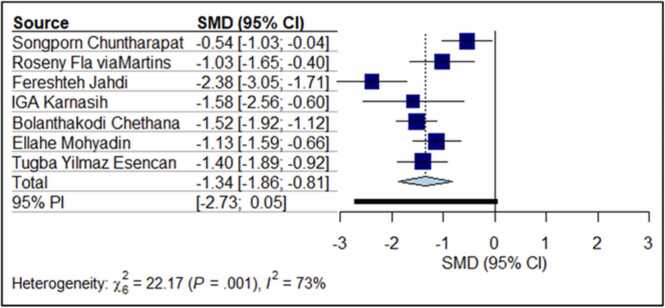
Effect of antenatal yoga on labour pain.

**Fig. 3:**
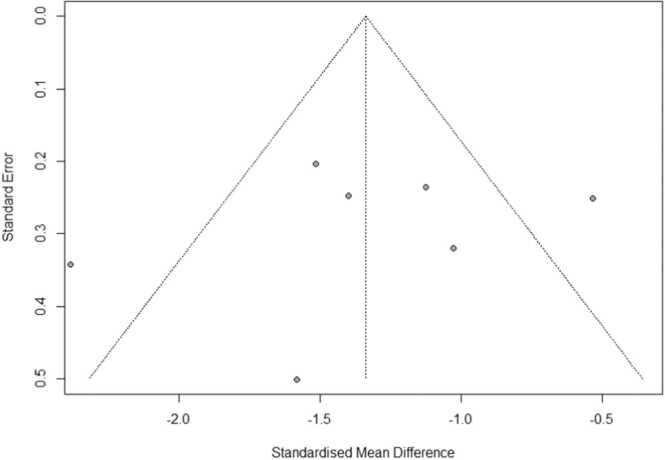
Funnel plot of the meta-analysis.

Seven RCTs with 462 participants reported the pain intensity through VAS. The pooled results (Fig. 2) showed that yoga is effective in reducing labour pain (SMD: −1.34 95% of CI: −1.86, −0.81) with significant heterogeneity among the studies (I^2^ =73%, p < 0.0008). Due to the small number of studies, we could not execute the initially planned sensitivity analysis. Findings from Egger test (p = 0.59) and symmetry of the funnel plot (Fig. 3) demonstrated that there is no significant publication bias exists in this study.

### Risk of bias assessment

**Fig. 4:**
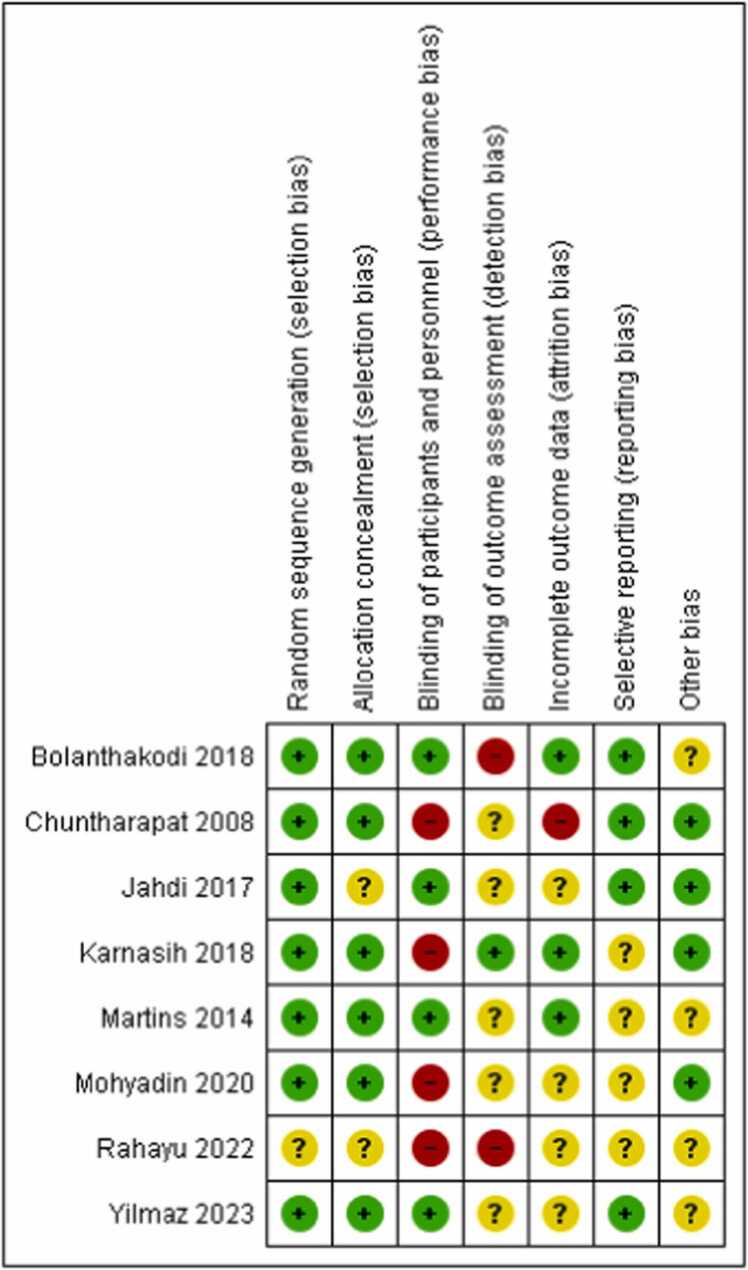
Risk of bias summary.

**Fig. 5:**
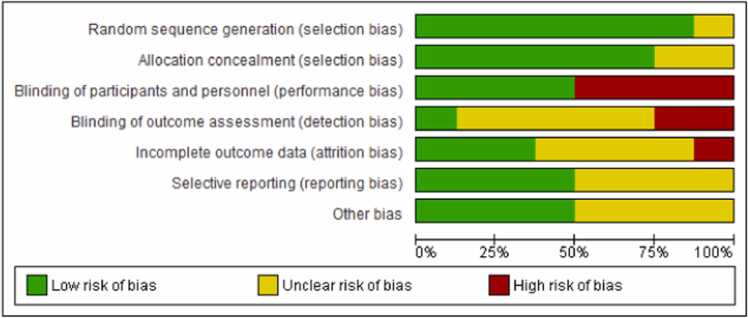
Risk of bias graph.

Majority of the studies rated low risk in random sequence generation, allocation concealment and other sources of biases. Majority of the studies rated high risk in blinding of participants and personnel reporting. More than half of the study rated low risk in incomplete outcome data. Regarding blinding of outcome assessment and selective reporting four studies had low risk and in rest of the studies it was unclear (Fig. 4, Fig. 5).

## Discussion

Yoga, a popular mind-body medicine, is frequently recommended for pregnant women. The aim of this systematic review and meta-analysis was to examine the published evidence on antenatal yoga, with a specific focus on its effectiveness in reducing the intensity of labour pain. The current systematic review and meta-analysis revealed evidence that antenatal yoga can reduce labour pain during delivery. In a recent meta-analysis investigating the effects of prenatal yoga on childbirth pain, five studies involving a total of 581 women were analyzed. The findings demonstrated a significant reduction in labour pain associated with prenatal yoga, as indicated by a standardized mean difference (SMD) value of −1.05 (95% confidence interval: −1.45 to −0.65; z = 5.15; P < 0.01). These results are consistent with our own findings, highlighting the beneficial impact of prenatal yoga in alleviating pain during labour [22]. However, that review only considered studies from PubMed, EMBASE, and Cochrane databases. Our systematic review, on the other hand, stands out by including studies from Web of Science, CINAHL, and Google Scholar. Moreover, we conducted a comprehensive literature search up to January 2023, ensuring that our review is up-to-date is one of the strengths of this current review. All the included studies provided at least one of these yogic interventions like asanas, pranayama, mudra, relaxation techniques and yoga nidra. However, the duration, frequency, outcomes and age groups varied widely. Through this meta-analysis, we postulate that yoga could be used as an effective non pharmacological intervention in the management of labour pain. Studies reported that practice of yoga reduces intensity of the labour pain, shortens the length of the labour, decreased number of caesarean section, oxytocin augmentation, usage of intravenous analgesics, improved rates of vaginal birth and tolerance to pain [23], [24]. Previous meta- analysis and systematic review on antenatal yoga found that yoga can reduce generalized body pain, lumbo-pelvic pain, rate of pre-term labour, intra-uterine growth retardation, low birth weight and pregnancy related complications [24], [25], [26]. Moreover, practice of yoga brings positive birth experience and greater satisfaction with pain relief. Antenatal yoga can also reduce stress, depression, and psychologically prepares the women for labour [27]. During pregnancy and labour, body undergoes several physiological and psychological changes[12]. Management of pain through yogic intervention involves both physical and psychological factors [28]. The practice of asanas strengthens the body and improves flexibility, in addition, asanas also influences secretion of hormone from endocrine gland [29], [30]. Practice of pranayama and breathing awareness brings mindfulness, regulates autonomic balance and also alleviates stress [23]. During the practice of yoga nidra and dhyana, their positive perception towards effective management of labour pain influences women in attaining better control over pain and also induces deeper state of relaxation [20], [31]. According to fear-tension-pain cycle, fear and tension can adversely affect the progress of labour and also increases the intensity of the labour pain [32]. Yoga can effectively manage pain by alleviating stress, fear and tension associated with labour. No adverse events were reported in any of the studies. However, due to the limited numbers of included studies, the relatively very small overall population, high heterogeneity no strong suggestions can be made regarding safety of the intervention.

Though this review revealed positive results for yoga, there are several limitations. Firstly, all the included studies displayed poor methodological quality. Secondly, as all studies used various forms/ style of yoga interventions, there was a lot of heterogeneity. Thirdly, study duration and frequency of the intervention vary between the individual studies. One more limitation is the usage of VAS, a subjective pain marker as the outcome measure. Despite the limitations, it cannot be ruled out that yoga has a positive influence in reducing labour pain. Since safety is more crucial for the evaluation of pain management with yoga intervention, future studies should address the safety aspects of the intervention as well more accurately.

## Conclusion

Yoga would be a promising mind –body intervention that can be used effectively to manage labour pain. We strongly recommend conducting robust randomized control trials with large sample size and appropriate protocol to confirm our findings. We also hypothesis that, prenatal yoga practise can reduce the number of augmentations of labour and caesarean sections.

## Ethical approval

Not applicable.

## CRediT authorship contribution statement

All authors have accepted responsibility for the entire content of this manuscript and approved its submission.

## Research funding

None declared.

## Declaration of Competing Interest

The authors declare that they have no known competing financial interests or personal relationships that could have appeared to influence the work reported in this paper.
